# Integrated Biotechnological Strategies for the Sustainability and Quality of Mediterranean Sea Bass (*Dicentrarchus labrax*) and Sea Bream (*Sparus aurata*)

**DOI:** 10.3390/foods14061020

**Published:** 2025-03-17

**Authors:** Sebastiano Rosati, Lucia Maiuro, Silvia Jane Lombardi, Nicolaia Iaffaldano, Michele Di Iorio, Michela Cariglia, Francesco Lopez, Martina Cofelice, Patrizio Tremonte, Elena Sorrentino

**Affiliations:** 1 Department of Agricultural, Environmental and Food Sciences (DiAAA), University of Molise, Via Francesco de Sanctis snc, 86100 Campobasso, Italy; s.rosati1@studenti.unimol.it (S.R.); nicolaia@unimol.it (N.I.); michele.diiorio@unimol.it (M.D.I.); lopez@unimol.it (F.L.); martina.cofelice@unimol.it (M.C.); tremonte@unimol.it (P.T.); sorrentino@unimol.it (E.S.); 2 Department of Agricultural, Forestry and Food Sciences (DISAFA), University of Turin, Largo Paolo Braccini 2, 10095 Grugliasco, Italy; 3 Gargano Pesca Società Agricola Consortile Arl-Società Benefit, Via Rucher 5, Interno 1/C, 71043 Manfredonia, Italy; cariglia.michela@gmail.com; 4 Gargano Shell Fish Farm Societa’ Cooperativa Agricola Arl, Pontile Alti Fondali, SC, 71043 Manfredonia, Italy

**Keywords:** Agenda 2030, aquaculture, sea bass, sea bream, biopreservation, edible coating, citrus waste, fish products

## Abstract

This review examines the current state of the supply chain management for *Dicentrarchus labrax* (sea bass) and *Sparus aurata* (sea bream), two key commercial fish species in the Mediterranean. It provides a comprehensive analysis of sustainable innovations in aquaculture, processing, and packaging, with particular attention to circular economy-based biopreservation techniques. A major focus is on the Integrated Multi-Trophic Aquaculture (IMTA) system, an advanced farming approach that enhances sustainability, promotes circular resource utilization, and improves fish welfare. By fostering ecological balance through the co-cultivation of multiple species, IMTA contributes to the overall quality of fish products for human consumption. Beyond aquaculture, the review addresses the critical challenge of food loss, which stems from the high perishability of fish during storage and processing. In this regard, it highlights recent advancements in biopreservation strategies, including the application of antagonistic microorganisms, their metabolites, and plant-derived extracts. Particular attention is given to the development of edible antimicrobial films, with a focus on the valorization of citrus processing by-products for their production. By centering on innovations specific to the Mediterranean context, this review underscores that a holistic, integrative approach to supply chain management is essential for transitioning the aquaculture sector toward greater efficiency and sustainability.

## 1. Introduction

A decade after the establishment of the 2030 Agenda for Sustainable Development, it is essential to assess progress in the management of key food production sectors, particularly in light of the projected global population increase of approximately two billion by 2050 [1]. The fisheries and aquaculture industries play a pivotal role in achieving several sustainable development goals (SDGs), as evidenced by extensive research demonstrating the relationship between sustainable management practices and broader environmental, economic, and social benefits [2,3,4,5,6]. Across diverse geographical regions, well-managed fisheries provide employment opportunities and economic stability, contributing directly to SDG 1 (No Poverty) [7,8,9]. Additionally, fish and seafood play an increasingly crucial role in global nutrition, supporting SDG 2 (Zero Hunger). According to the Food and Agriculture Organization of the United Nations (FAO), fish and fishery products account for approximately 20% of animal protein consumed worldwide, with their intake growing faster than other protein sources [10]. Beyond their nutritional contribution, fish and seafood are highly valued for their sensory attributes and their role in a balanced diet, offering essential proteins, vitamins, and long-chain polyunsaturated fatty acids (PUFAs), including omega-3 [11,12,13,14]. Consequently, aquaculture-derived fish products not only contribute to global food security but also support SDG 3 (Good Health and Well-being).

Driven by rising demand, the fisheries and aquaculture sectors have experienced exponential growth, positioning them among the fastest-growing food industries, particularly in developed nations. Between 1960 and 2022, global fish production for human consumption increased from 22 million to 223 million tons, with aquaculture contributing 52% of total production, a figure expected to rise by 20% by 2030 [15,16,17].

**Figure 1:**
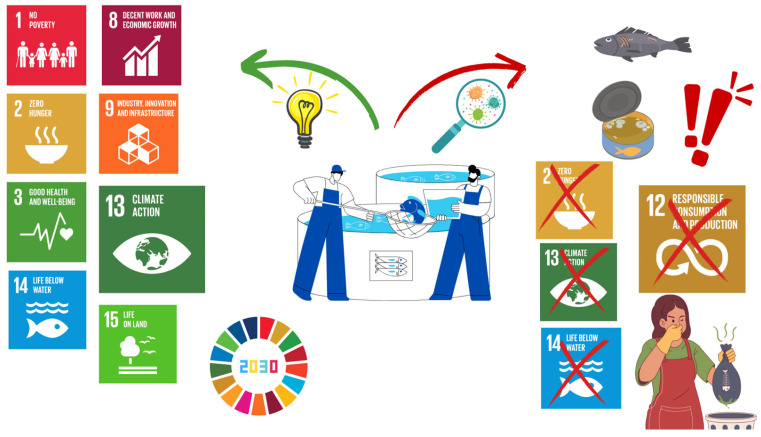
Relationship between aquaculture supply chain management and the achievement of the Agenda 2030 Sustainable Development Goals.

**Figure 2:**
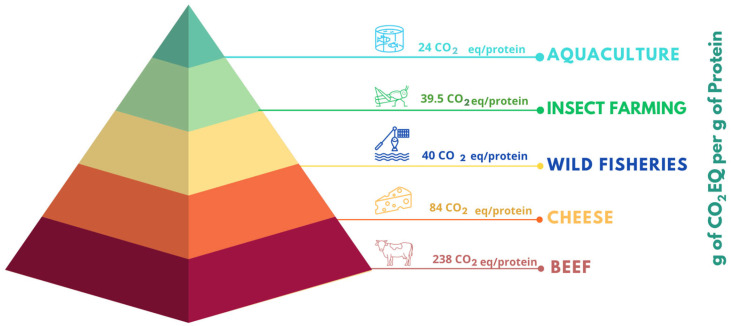
Greenhouse gas emissions expressed in grams of CO_2_ equivalent per gram of proteins [30,32].

In the Mediterranean region, aquaculture has witnessed consistent expansion due to favorable environmental conditions, including optimal water temperature and salinity. The industry is predominantly centered around *Sparus aurata* (gilthead sea bream) and *Dicentrarchus labrax* (European sea bass), which collectively reached a production volume of 520,000 tons in 2022. Notably, Mediterranean countries account for 97% of global sea bass and sea bream production, highlighting the region’s significance in the global market [18,19]. However, as the sector approaches the 2030 SDG targets (Figure 1), it faces significant environmental challenges. Intensive aquaculture operations frequently introduce disinfectants, veterinary pharmaceuticals, and residual feed into marine ecosystems, leading to nutrient pollution, oxygen depletion, and harmful algal blooms. In response, the industry has increasingly embraced circular economy principles aimed at reducing environmental impacts by optimizing resource utilization and minimizing waste [20]. Circular economy strategies in aquaculture encompass: (i) improved waste management through wastewater reuse and seafood by-product valorization [21,22], (ii) innovative feed formulations and farming systems that repurpose excess nutrients across trophic levels [23,24], (iii) economic diversification via novel products and markets [25], and (iv) enhanced conservation efforts that benefit marine biodiversity and coastal communities [26]. Additionally, adopting sustainable aquaculture practices requires regulating nutrient cycling and carbon sequestration to mitigate ecosystem degradation [27]. The economic and social dimensions of fisheries and aquaculture further align with SDG 8 (Decent Work and Economic Growth) and SDG 9 (Industry, Innovation, and Infrastructure) by fostering employment, technological advancement, and equitable trade practices. Moreover, sustainable aquaculture directly contributes to SDGs 14 (Life Below Water) and 15 (Life on Land) by promoting biodiversity conservation and reducing overfishing pressures [4,28,29]. Importantly, aquaculture aligns with SDG 13 (Climate Action) by offering a low-carbon alternative to terrestrial livestock. Comparative studies indicate that the CO₂-equivalent emissions of wild-caught fish are approximately six times lower than beef, more than twice as low as cheese, and nearly equivalent to insect protein. Aquaculture products demonstrate even lower emissions—approximately 10 times lower than beef and nearly half that of insect farming (Figure 2) [30,31].

Despite these advantages, the Mediterranean fish supply chain remains challenged by overfishing, illegal, unreported, and unregulated (IUU) fishing, aquaculture-related environmental impacts, and climate change-induced ecosystem disruptions. Additionally, food loss and waste along the fish supply chain undermine sustainability objectives, particularly SDG 12 (Responsible Consumption and Production). Addressing these challenges necessitates integrating eco-friendly packaging solutions [33,34] and minimally processed foods free from chemical additives [35] in alignment with the European Green Deal’s objective to achieve climate neutrality by 2050. Offshore aquaculture, while alleviating pressure on wild stocks, must enhance cold chain logistics and eco-friendly preservation techniques to mitigate waste and environmental footprint. This review examines the innovations shaping the Mediterranean aquaculture supply chain, with a specific focus on *Sparus aurata* and *Dicentrarchus labrax*, the two most commercially relevant species in the region [36,37]. It explores advancements in sustainable aquaculture, including Integrated Multi-Trophic Aquaculture (IMTA) systems, which optimize resource efficiency through trophic interactions. Additionally, the review discusses biopreservation techniques, such as edible antimicrobial films derived from citrus by-products, illustrating the intersection of aquaculture with circular economy principles. By consolidating the latest scientific and technological advancements, this review underscores the critical need for a holistic, integrative approach to fisheries and aquaculture to achieve long-term sustainability and economic viability.

## 2. Quantitative Research Literature Analysis

Quantitative bibliometric analysis is a widely used analysis of large volumes of scientific data, which allows articles to be sorted according to document type and source, year of publication, and subject area, and allows the most popular items and the relationships between them to be visualized. Specifically, in this review, the analysis aimed to reveal the subject areas covered by the largest number of publications and also to identify research perspectives in relation to innovations in the supply chain of the two fish species. Firstly, the analysis focused on the association between aquaculture in the Mediterranean area and food preservation. The results of this study could provide an overview of a bibliometric review of the literature on the integrated supply chain view of the two Mediterranean fish species. Secondly, the research reveals the subject areas of most of the publications and shows the trend of studies related to solutions and innovations in the supply chain. In our research, the data used for bibliometric analysis were retrieved from Scopus and visualized using the similarity viewer (VOS), which aims to provide easy formation and visualization of bibliometric maps.

This method enables the efficient collection of literature and the establishment of interrelationships between the chosen publications within the options. The set of keywords, including sea bass, sea bream, Mediterranean area, food preservation, and aquaculture, returned 170 papers retrieved from the Scopus database limited to the year of publication, 2010–2025. The results received were downloaded in RIS format to be processed with VOSviewer to visualize and analyze trends in bibliometric form. VOSviewer makes it possible to create network-based (co-occurrence) country maps, build a keyword map based on shared networks, and create maps with many elements [38,39]. All keywords that contributed to the full count method were considered as an analysis element during co-occurrence mapping. To achieve a more accurate result, the study placed certain constraints on the analysis. The number of keywords to be used can be adjusted by eliminating less relevant keywords. A minimum of five occurrences of a keyword was applied as a limiting factor.

**Figure 3:**
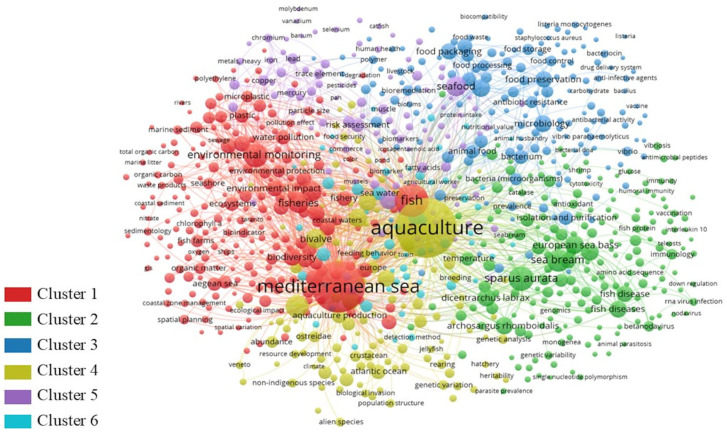
VOS visualization of the co-occurrences map between the selected keywords (aquaculture, Mediterranean area, spoilage, food preservation) processed in VOSviewer software version 1.6.20.

The map (Figure 3) shows the number of occurrences of the term in the article and the relationship between the keywords. In the network, each term is presented by a circle, while the size of the circle is commensurate with the number of publications in which the term is found. Each color represents a cluster of items, and the size of the curved lines specifies the approximate connection of the items’ repetition, while the thickness of the curved lines indicates the strength of the relationship between pairs of subject areas or between items. The red cluster gathers the largest number of items (222), among which the Mediterranean Sea is the most recurrent entry. The cluster mainly gathers topics related to fishing in the Mediterranean area and environmental aspects, from pollution to biodiversity. The green cluster groups 192 items related to fish species and their biodiversity. It is evident that sea bass and sea bream are the most present species. The blue cluster gathers 158 items, mainly concerning microbiological issues and the packaging of fish products. The yellow cluster, although not the most numerous, contains terms with the highest number of occurrences, such as aquaculture. Furthermore, the yellow cluster, and in particular the entry aquaculture, establishes the most relationships with all the other clusters. Items mainly related to health aspects are grouped in the purple cluster. Finally, the blue cluster gathers less than 15 items that establish few and tenuous relationships with the other clusters.

## 3. Technological Systems for Sea Bass (*Dicentrarchus labrax*) and Sea Bream (*Sparus aurata*) Farming in the Mediterranean Area

Sea bass and sea bream are among the most farmed fish in the Mediterranean due to their high market demand, contributing significantly to both local economies and global seafood markets [18]. Driving consumer preferences are their mild flavor, firm texture, and rich nutritional value (rich in omega-3 fatty acids, proteins, and essential nutrients), while their fast growth and adaptability make them ideal for aquaculture.

Over the past few decades, this sector has experienced continuous expansion, driven by advancements in hatchery technologies, selective breeding, and improved feed formulations, which have enhanced growth rates and feed conversion efficiency.

Farming these species supports economic stability, coastal employment, and sustainability by reducing pressure on wild stocks and enhancing food security in the region [18,40,41].

Despite these advances, the industry faces ongoing challenges, such as disease outbreaks, market fluctuations, and regulatory pressures aimed at minimizing environmental impact. The most common farming systems include sea cages, land-based tanks, and raceways. Sea cage farming is the predominant method, especially in coastal areas with suitable environmental conditions such as calm waters and optimal temperatures. These cages allow for the large-scale production of fish in their natural environment, promoting efficient water exchange and providing a cost-effective solution for farmers. However, sea cages raise environmental concerns related to feed and energy consumption, the release of nutrients and organic compounds into the water, the use of pesticides and antibiotics [41], and interactions with wild populations.

The two most used cage systems are inshore and offshore cages, which differ primarily in their location relative to the coast and the environmental conditions they provide. In shore cages are located near the coast, in sheltered bays, lagoons, or shallow marine areas. Offshore cages are in the open sea, at a significant distance from the coast.

Land-based systems, such as tanks and raceways, offer greater control over environmental parameters, reduce the risk of escapes and interactions with wild fish. However, these systems entail higher operating costs by providing treatment infrastructure, greater control over critical parameters such as oxygenation levels, temperature, and consequently a greater environmental impact [42,43].

Recirculating aquaculture systems (RAS) allow optimal control of environmental parameters while reducing water use and waste production [44]. Despite the high initial investment and operational complexity, RAS systems are considered a promising solution for sustainable aquaculture.

Each of these systems has been tailored to meet the specific needs of the Mediterranean aquaculture industry, balancing productivity with environmental protection. Ongoing research and technological innovation continue to refine these systems to improve their sustainability and economic viability.

Environmental concerns, particularly those related to nutrient discharge, habitat alteration, and the use of antibiotics, have prompted the development of more sustainable strategies to improve farming conditions and fish welfare, such as the use of alternative feeds containing insects and algae and precision aquaculture with sensors and AI to track water quality and fish health, reducing stress and diseases [45]. Among other strategies, one approach gaining attention is the Integrated Multi-Trophic Aquaculture (IMTA) system, which aims to mimic natural ecosystems by cultivating species from different trophic levels together [46,47].

### 3.1. Integrated Multi-Trophic Aquaculture (IMTA)

IMTA is an innovative and sustainable farming system that leverages the synergistic cultivation of different aquatic species across various levels of the food chain. This approach replicates natural ecosystems by converting waste products from fed species like fish or shrimp into valuable inputs for other organisms. Organic extractive species, including shellfish, herbivorous fish, and echinoderms, utilize uneaten feed and feces, while inorganic extractive species like seaweed absorb dissolved nutrients [48,49]. This cyclical design reduces environmental impact, enhances system efficiency, and fosters a holistic approach to aquaculture. Extensive research has highlighted IMTA’s potential to improve the ecological sustainability of aquaculture [30,50,51,52,53,54,55,56], economic [25], and social sustainability [26] in fish farming. Compared to monoculture systems, IMTA has been shown to significantly influence microbial community composition and dynamics, with biotic factors (e.g., the presence of species like shrimp) and abiotic factors (e.g., nutrient levels in the water) shaping microbial populations [57]. These microbial communities play a crucial role in both nutrient cycling and shaping the intestinal bacterial communities of cultured species, influencing key physiological processes such as metabolism, nutrition, and immune system development [58,59]. Studying these microbial communities during the culture process can provide valuable insights into their interactions with cultured species, helping to optimize culture methods, expand production, and increase profitability. Moreover, studies have shed light on the impact of bacterial communities on the presence of antibiotic-resistance genes (ARGs). While ARGs were detected throughout the culture process, their relative abundance decreased over time in some IMTA systems, suggesting a potential removal effect by microbial communities [57].

### 3.2. IMTA for Sea Bass and Sea Bream Farming

In summary, IMTA not only improves the ecological balance of aquaculture systems but also plays an important role in managing microbial communities and reducing the spread of antibiotic resistance, thus contributing to the environmental sustainability of aquaculture. This feature underscores IMTA’s role in promoting sustainable and responsible aquaculture practices. IMTA’s advantages are particularly noteworthy when applied to Mediterranean species such as European sea bass (*Dicentrarchus labrax*) and gilthead sea bream (*Sparus aurata*), IMTA presents several key advantages. Though research on sea bass in IMTA systems is still limited, the existing studies suggest promising outcomes, particularly in terms of growth performance, environmental sustainability, and system efficiency. For gilthead sea bream, the body of evidence is more robust, showing clear benefits, including improved growth rates, better resource utilization, and enhanced system stability [60,61]. The integration of these species into IMTA systems has led to significant improvements in their zootechnical performance. In particular, sea bream has demonstrated superior growth in IMTA systems compared to conventional intensive or semi-intensive systems. This improvement is largely attributed to improved water quality and nutrient availability facilitated by co-cultivated species such as seaweed and shellfish. These species act as biofilters, absorbing excess nutrients and reducing waste, thereby creating a balanced ecosystem that promotes fish health and faster growth rates. Although studies on sea bass are limited, preliminary results suggest similar benefits mirroring outcomes observed in other species under comparable conditions. In terms of sensory attributes, IMTA-raised sea bream exhibits unique features that distinguish it from fish produced in monoculture systems. Notably, the sea bream from IMTA systems shows more intense physical features, such as an intensified interorbital yellow band and a brighter orange patch on the gill cover. The flesh also appeared darker and slightly yellowish, likely due to the fish’s consumption of algae. Additionally, the odor profile is marked by pronounced marine and iodine notes, which may be attributed to the influence of species like Ulva in the IMTA ponds [62]. Nutritional benefits are another key aspect of IMTA. Sea bream raised in this system has higher levels of omega-3s, including docosahexaenoic acid (DHA) and eicosapentaenoic acid (EPA). The nutrient-rich environment fostered by IMTA allows for better utilization of resources, enhancing the overall health and quality of the fish. Animal welfare is another critical area where IMTA provides benefits. The more natural and balanced environment of IMTA systems generally reduces stress levels in fish, leading to improved health and welfare. This balanced environment supports physiological stability, disease resistance, and general well-being, which are critical for aquaculture productivity. Although there is limited research specifically focused on the welfare of sea bass and sea bream in IMTA systems, the overall improvements in water quality and ecosystem balance suggest that these species would experience similar welfare benefits, such as reduced stress and better overall condition. The integration of European sea bass and gilthead sea bream into IMTA systems offers numerous advantages. These include enhanced growth performance, improved sensory qualities, superior nutritional content, and better animal welfare, all while supporting environmental sustainability [61]. Given these promising outcomes, it is clear that IMTA represents a sustainable and economically viable alternative to traditional monoculture systems. However, further research on the production of sea bass and sea bream in IMTA systems is crucial to fully explore and optimize their potential within this innovative framework. Understanding the specific interactions between these species and their environment in IMTA settings could unlock new possibilities for aquaculture in the Mediterranean area.

## 4. Sea Bream and Sea Bass Microbiota

**Figure 4:**
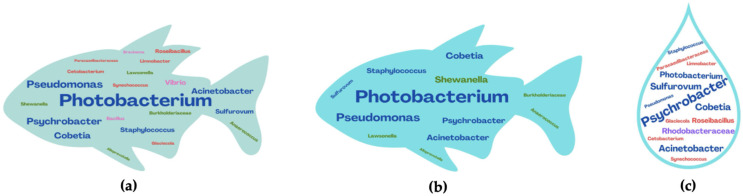
Microbiota of sea bream gut (**a**) and skin (**b**), and seawater (**c**). The font size of each genus varies according to its frequency. The genera with blue color are those present in all three environments (**a**–**c**); the green ones are only in (**a**,**b**); the red ones in (**a**,**c**). Those present only in the gut (**a**) are fuchsia, and those present only in seawater (**c**) are purple. The figure was elaborated with the data of Quero et al. [65].

The microbiome of fish species profoundly influences host biochemical events and plays a significant role in commercially valuable species by interfering with the shelf-life, healthiness, and quality of the resulting food products. Each species of aquatic organism is characterized by a specific microbiota, shaped by the type of water (seawater or freshwater), geographical origin, water contamination, and season of capture [11,62]. The relationship between environmental and fish microbiomes is quite well investigated [63,64]. Different authors have also shown that microbiome-based approaches can be adopted as a tool to trace the origin of seafood products. Investigating the microbiota of sea bass and sea bream in the Mediterranean area, distinctions between the fish microbiomes and those of the surrounding environment, as well as between different species, have been highlighted (Figure 4) [65,66,67].

At the same time, however, the connection between the microbiota of fish tissues and gut and that of the environment has been highlighted.

This microbiota is mainly concentrated on the skin and in the digestive tract and gills in highly variable concentrations between 2 log cfu and 9 log cfu/g or cm^2^. In the intestine, there is a concentration of facultative and strict anaerobes, while the skin and gills host aerobic psychrophilic and psychrotrophic bacteria [11,68]. As long as the animal is alive, the muscles are sterile and free of microorganisms that do not invade them thanks to the protection of natural defenses, but when, after capture, the fish dies, the microorganisms can enter the muscles, contaminating them [11]. Halotolerant *Psychrobacter* spp. *Anaerococcus* spp., and *Vibrio* spp., are bacteria generally found in both sea bass and sea bream farmed in the Mediterranean area (Figure 4). Acinetobacter, *Psychrobacter*, and Shewanella are mainly present on the skin of the fish. Lawsonella, *Staphylococcus*, Pseudomonas, and Cutibacterium have been found to populate the microbiota of the intestine, gills, and tissues. Furthermore, Firmicutes (*Anaerococcus* spp., *Staphylococcus* spp.) and Actinobacteria (e.g., *Cutibacterium*) are generally recognized as the dominant phyla in the gut microbiome [65,69].

Bacteroidetes, which are generally found in abundance in aquatic farm environments, are sparsely present in the fish microbiota. This is because these bacteria dominate the microbiota of herbivorous fish and not carnivorous fish. However, it should be noted that the microbiota of sea bream and sea bass, although presenting differential taxa compared to those of the breeding environment, also includes several overlapping microorganisms [70]. Studies in recent years [65], although to be further explored, highlight complex interactions between the microbiota of the two fish species and the microbiota of the surrounding environments. Knowledge of these relationships can be adopted to improve aquaculture systems. Some authors [71] have highlighted that the IMTA system avoided the bloom of cyanobacteria and allowed the abundance of beneficial microorganisms in the sediments. However, the specific topic, when also considering its importance in facilitating the achievement of several SDGs of Agenda 2030, is that future investigations are needed.

**Figure 5:**
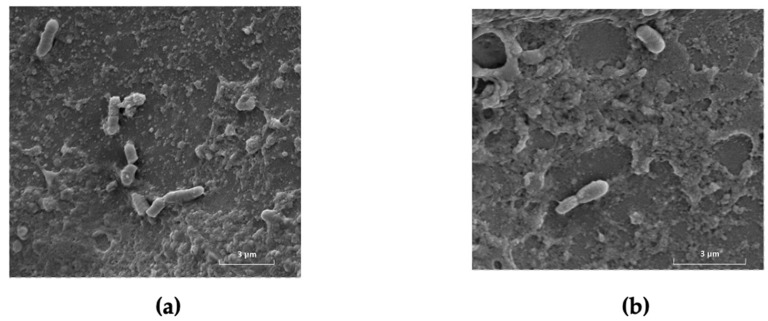
Scanning electron microscope (SEM) photo of microbial cells present on sea bass skin samples.

In the framework of some ongoing research projects at the Department of Agricultural, Environmental and Food Sciences of the University of Molise, preliminary analyses were performed to evaluate the microbiological quality of sea bream and sea bass from an integrated multi-trophic aquaculture system farming located in Puglia (Italy). The farm is an offshore facility with floating cages, each measuring 20 m in diameter and 8 m in net depth, anchored to a seabed 10 m deep. Each cage holds approximately 100,000 animals, providing ample space for natural movement while ensuring appropriate density. The system promotes optimal water circulation, supporting the health and growth of the fish in a sustainable, antibiotic-free environment. The microbiological analyses, carried out, according to Reale et al. [72], at the food microbiology laboratory of the Department of Agricultural, Environmental and Food Sciences (DiAAA) of the University of Molise, highlighted undetectable contamination by the pathogens and SSOs studied in all the sea bream and sea bass samples analyzed. The TVC (total viable microbial counts) and psychrophilic microorganisms present on the skin of the analyzed sea bream and sea bass showed concentrations of about 2 log/CFU, data consistent with those reported in the literature [73]. The Figure 5a,b obtained by observing the skin of sea bass and sea bream with a scanning electron microscope (SEM) (Tescan Vega 4), located in the Microscopy Center of the DiAAA Department (University of Molise), confirmed the presence of a low number of bacterial cells on the skin of the fish analyzed. SEM observation proved to be particularly useful because it allowed us to identify the site where the microorganisms live in the area delimited by the scales and the skin.

In recent years, innovative microbial identification methods have been developed, mainly high throughput sequencing (HTS) techniques, which have revolutionized the study of seafood microbiota, offering deeper insights into its composition and dynamics [13]. These advanced methods have enabled researchers to identify microbial communities with greater accuracy, shedding light on spoilage mechanisms and potential interventions to improve seafood quality and safety. For instance, Parlapani et al. [73] reported high concentrations of the genera *Psychrobacter* and Carnobacterium in sea bream from the Ionian and Aegean seas during storage at 8 °C. In addition, Pseudomonas was identified as a prevalent genus in sea bream from the Ionian Sea, highlighting its significant role in microbial spoilage during storage.

Fish products are widely regarded as highly perishable foods due to their nutrient-rich composition, which makes them susceptible to microbial growth. The shelf-life and safety of fish products are influenced by a range of factors, including raw material quality, time elapsed from capture to sale, storage temperatures and handling, processing, packaging, and distribution methods [74]. All these factors, in fact, can influence the microbiological quality of fish products, directly impacting spoilage rates and safety [75].

In addition, these products are still often preserved by traditional methods such as refrigeration, freezing, drying, fermentation, salting, and canning. In particular, refrigeration, which is one of the most used methods worldwide, allows to inhibit many pathogenic microorganisms, which are unable to grow at low temperatures. However, this technology, used alone, is not adequate to inhibit psychrotrophic microorganisms, among which there are also pathogenic and spoilage species [76]. During storage, fish undergo microbial deterioration caused by SSOs (specific spoilage organisms), a subset of the initial microbiota capable of outcompeting other microorganisms and reaching high concentrations. These SSOs produce metabolites, such as trimethylamine (TMA), ammonia, and volatile sulfur compounds, responsible for off-flavors and odors, signaling spoilage [75,76,77].

Both sea bass and sea bream are susceptible to microbial growth. SSOs mainly degrade proteins and lipids, leading to structural breakdown, textural changes, and a decline in sensory quality, ultimately rendering the product unfit for consumption [78].

### 4.1. Potential Pathogenic Microorganisms

The European Food Safety Authority (EFSA) [79] and European Centre for Disease Prevention and Control (ECDC) reported that, in 2023, “fish and fishery products” ranked as the fourth most frequent food vehicle associated with foodborne outbreaks with strong evidence across EU member states. Additionally, the consumption of this kind of food was associated with the highest number of deaths among cases in strong-evidence outbreaks (seven deaths). A wide range of causative agents has been associated with outbreaks caused by “fish and fish products”, including in decreasing order histamine and scombrotoxin, *Salmonella*, marine biotoxins, *Bacillus cereus* toxins, norovirus, *Listeria monocytogenes*, *Staphylococcus aureus* toxins, *Campylobacter*, *Shigella*, *Clostridium perfringens* toxins, and *Clostridium botulinum* toxins. As regards *L. monocytogenes*, “fish and fishery products” were found to be one of the food categories most frequently contaminated by this pathogen [78]. This foodborne pathogen poses a significant risk as it causes listeriosis, a severe foodborne disease characterized by high hospitalization and mortality rates, particularly among vulnerable populations such as pregnant women, the elderly, and immunocompromised individuals [80]. In 2023, listeriosis and West Nile virus infection were the two most severe diseases in the EU, with the highest rates of mortality and hospitalization among reported cases. Almost all listeriosis cases with available data were hospitalized (96.5% of confirmed cases). As in recent years, the highest number of deaths was associated with listeriosis (N = 335), followed by salmonellosis (N = 88) and West Nile virus infection (N = 75). Listeriosis and West Nile virus infection were also the zoonoses with the highest mortality rates, 19.7% and 11.2%, respectively [79]. According to Eissa and Younes [81], the high incidence of L. monocytogenes in fish products is primarily attributed to their common marketing as refrigerated or fresh items, conditions that can facilitate microbial contamination and growth. Interestingly, L. monocytogenes is not a native microorganism in marine environments [82]. Lyautey et al. [83] suggest that contamination may occur through agricultural runoff containing irrigation water or animal feces entering the marine ecosystem. Furthermore, improper post-harvest handling practices, such as delayed evisceration, can lead to cross-contamination, as L. monocytogenes from the intestines can spread to other parts of the fish [84]. Additionally, contamination risks are heightened by inadequate hygiene practices during processing, such as the use of contaminated tools or improper handling by operators [85]. The concern about Listeria is also due to its ability to resist and grow in different environmental conditions, even overcoming the technological treatments provided for the preparation of ready-to-eat products [86,87]. Fish products, including those from species like sea bass (*Dicentrarchus labrax*) and sea bream (*Sparus aurata*), can harbor various pathogens, such C. botulinum and human-origin pathogens like *Salmonella* spp. and *E. coli*, *Staphylococcus aureus*. Environmental contamination during processing and storage introduces additional risks, including *Bacillus cereus*, *C. perfringens*, and *L. monocytogenes*. Arab et coll. [88] conducted a comprehensive assessment of *Vibrio* spp. occurrence in 690 samples of wild and farmed sea bass and sea bream collected along the Algerian Mediterranean coast. The overall incidence of *Vibrio* spp. across all samples was 6.08%. However, the rate was higher among farmed fish, reaching 7.92%. Alarmingly, *Vibrio cholerae*, a highly pathogenic species responsible for severe foodborne epidemics, was detected in 2.83% of the farmed fish samples. This finding underscores the need for stringent monitoring and control measures, particularly in aquaculture environments where contamination risks may be amplified by farming practices and environmental factors.

### 4.2. Spoilage Microorganisms

**Figure 6:**
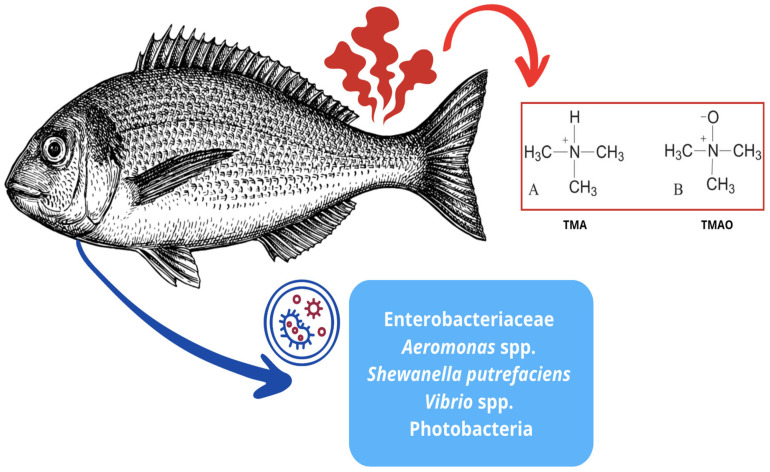
Microorganisms in fish products with enzymatic action are capable of converting TMAO (trimethylamine oxide) to TMA (trimethylamine), which causes a particular smell of spoiled fish.

The deterioration of fish products is due to the action of several factors, including endogenous enzymatic activities and, above all, the development and metabolic activities of microorganisms, which lead to the decay of the product within a few hours of capture [13,75]. As described by Suresh and colleagues [89], the quality of fish products is perceived through the different senses; in particular, the consumer evaluates the sensorial and organoleptic characteristics, focusing on smell, flavor, appearance, and texture. Indeed, the smell of fresh fish is pleasant for the consumer, reminiscent of the smell of the sea, but if the storage of this product is prolonged and, above all, unsuitable, it can lead to the formation of unpleasant odors. Generally, fish is considered a highly perishable food immediately after capture as several degradative mechanisms come into play, such as post-mortem enzymatic autolysis, lipid oxidation, and antimicrobial action with subsequent spoilage. The first degradation phenomenon that occurs in fish is the activation of autolytic enzymes within the muscle that leads to rapid protein degradation and the formation of biogenic amines by decarboxylase. The unpleasant odor is due to the formation of unwanted compounds due to microbial growth in the product. The compounds formed are trimethylamine (TMA), dimethylamine (DMA), volatile sulfur compounds, and total volatile basic nitrogen (TVBN) [90]. It is known that there are not only low-fat fish species; therefore, especially in fish with a high-fat content (for example, salmon), there is a high possibility of oxidation of lipids (especially unsaturated fatty acids) with the formation of peroxides, aldehydes and more. Various types of bacteria, including *Shewanella putrefaciens*, *Aeromonas* spp., *Photobacterium phosphoreum*, *Vibrio* spp., and others utilize TMAO (trimethylamine oxide) to regulate dehydration in the marine environment by converting it to TMA (trimethylamine), which causes ammonia-like bad tastes (Figure 6) [91].

The main causes of the rapid deterioration of fish, including sea bass (*Dicentrarchus labrax*) and sea bream (*Sparus aurata*), are closely linked to some of its characteristics, such as high-water activity and low acidity levels, which are responsible for the rapid proliferation of bacteria [13]. As described by Alasalvar et al. [92], following death, fish are subject to microbial contamination of the gills, skin, and intestines. All this happens in unsuitable storage conditions, so the storage temperature is a fundamental requirement for good preservation of the product, thus guaranteeing safety of use [11]. As is known, the use of cold or ice allows for limiting microbial proliferation, thanks to the bacteriostatic action this has on the product [93]. Both farmed and wild-caught sea bass and sea bream often face significant food safety challenges due to poor microbiological and hygienic quality, leading to high quantities of unused fish. Among the various microorganisms that spoil fish products, Psychrobacter is found, in addition, *Pseudomonas* and *Shewanella* have already been confirmed several times [90]. As reported by Rosado et al. [94], the Pseudomonadota (formerly Proteobacteria) and Bacteroidota (formerly Bacteroidetes) (NCBI Taxonomy) individually represent 50% of the microorganisms present in the gills and skin of sea bass and sea bream. During storage, spoilage bacteria grow faster than other components of microbiota, producing compounds that cause unpleasant odors and a reduction in sensory quality. They hydrolyze proteins into peptides and amino acids, this hydrolysis causes profound changes in fish flesh by altering texture, color, water-holding capacity and other physicochemical properties.

To evaluate the freshness of fish, specific tests are being developed to identify bacteria such as *Shewanella putrefaciens*, *Photobacterium phosphoreum*, and *Pseudomonas* spp., which are indicative of deterioration more precisely. Although these tests provide reliable data, they require an incubation period that can vary from a few hours to several days. For these reasons, the scientific community, using sequencing (NGS) and PCR (polymerase chain reaction) techniques, can identify deteriorating and pathogenic microorganisms and, once identified, apply the necessary precautions to avoid their deteriorating action on the product [62]. Conducting research and studies on developments in the microbial profile of seafood products and the aquatic environment will provide a greater understanding of how new food technologies can contribute to the increased shelf life of these easily perishable products [13].

### 4.3. Useful Microorganisms and Biopreservation

As previously discussed, the proliferation of undesirable microorganisms is a significant factor contributing to the decline in the sensory and nutritional quality of fish products, ultimately leading to food spoilage and waste. This issue has broad implications, particularly as it undermines efforts to achieve sustainable development goals (SDGs), such as ensuring sustainable consumption and production patterns (Goal 12) and reducing food waste. With the growing consumer preference for minimally processed, additive-free products and global commitments like the 2030 Agenda, biopreservation has gained prominence as a sustainable and effective approach to mitigating these challenges.

Biopreservation strategies to improve the safety and extend the shelf-life of perishable food products utilize protective microbial cultures [86,95], microbial metabolites [80], or natural compounds of plant origin [76,96,97,98]. This approach aligns with clean-label trends, catering to consumers’ preference for natural, healthy food products while supporting the UN’s Sustainable Development Goals by reducing food waste and improving resource efficiency [95].

The concept of biopreservation, which refers to the use of antagonistic microorganisms and their metabolites, mainly involves the use of lactic acid bacteria (LAB), which are recognized as safe organisms, resistant to various environmental conditions, often capable of interacting harmoniously with other useful microorganisms, and producers of numerous metabolites with antimicrobial action [86,98,99,100]. LAB competes with and/or exerts antimicrobial activity against undesirable microbiota. But while LAB shows strong antagonistic or competitive activity against Gram-positive pathogens such as *L. monocytogenes*, they are less effective against Gram-negative SSOs such as *Pseudomonas* spp., *Shewanella putrefaciens*, and *Photobacterium phosphoreum* [95].

Microbial metabolites have gained increasing attention as natural and sustainable tools for the preservation of fish and fishery products. These metabolites, particularly those produced by LAB, offer significant advantages in combating spoilage and pathogenic microorganisms, thereby extending shelf life and enhancing food safety. LAB metabolites, such as organic acids (e.g., lactic and acetic acids), lower the pH of the product, creating an environment unfavorable to microbial growth.

Additionally, bacteriocins, bioactive peptides with bacteriostatic or bactericidal properties, have demonstrated efficacy, particularly against Gram-positive microorganisms. Other metabolites, including reuterin, diacetyl, and hydrogen peroxide, further enhance antimicrobial action by targeting diverse microbial cell structures and functions [101]. Despite these advancements, there are some critical issues in the use of LAB metabolites; in fact, they can cause sensorial changes in the product due, for example, to the lowering of pH due to organic acids, highlighting the need for a careful application to maintain the desirable taste and texture of fish products [102]. Furthermore, storage conditions, fish species, and metabolite concentrations influence the efficacy of these compounds, highlighting the complexity of their practical application [101]. For these reasons, it will be necessary to focus on the action of LAB for the conservation of fish products with further studies. Some scientific studies, however, have demonstrated their effectiveness in fish products.

In addition to the use of specific microorganisms for biopreservation activity, several studies have also demonstrated the use of lytic bacteriophages for food preservation [103]. Their peculiarity is that they are highly specific for a given microbial species without altering the microbial and sensorial characteristics of the food product [104]. Their use is diversified in different foods, as bacteriophages can be stable at different pH and temperature conditions [105]. Yang et al. [106] have demonstrated how the use of a lytic phage (SPMIX3-156) can block the growth of Shewanella baltica in catfish at refrigeration temperature and that the bacteriophage PD1 inhibits the growth of *Pseudomonas fluorescens* in refrigerated grass carp, having positive effects on the shelf life of the product [107]. However, the use of bacteriophages on sea bass and sea bream fillets has not yet been demonstrated in the literature, so it would be useful to investigate the issue further by testing these on the samples of interest to us.

Recent studies have focused on natural extracts derived from plant matrices, particularly for their antimicrobial and antioxidant properties [96,97]. These extracts are rich in phenolic compounds, known for their ability to disrupt microbial cell membranes and interfere with essential enzymatic functions [76]. Such natural solutions that align with consumer demands for clean-label products while enhancing the shelf life and safety of fish and seafood products will be discussed in the next chapter.

## 5. Food Losses and Waste in the Fish Supply Chain

Food waste, according to FAO [108], is defined as the withdrawal of food or inedible portions of food from the food chain, but also how these are intentionally left to spoil or expire. Food losses and waste may occur at any point along the food chain. According to Kaza et al. [109], solid waste output reached 2.01 billion tons globally in 2016, and it is predicted that 3.4 tons of waste will be produced by 2050.

Losses and waste in the fisheries sector, estimated at between 30% and 50% of total production [68,110], represent a global problem that severely undermines SDG 12 (Sustainable Consumption and Production). Large amounts of fish products are lost along the supply chain, significantly impacting the sustainability of this sector. In particular, a large portion of losses occur during fishing, when unwanted fish or parts of caught fish are discarded and thrown back into the water [111]. Furthermore, fish products are often caught at great distances from the area where they will be processed or marketed without having conservation methods that prevent their rapid spoilage.

In contrast to other supply chains, the primary cause of losses in the fish supply chain is the high perishability of fresh products rather than strained relationships between supply chain participants. In fact, due to their specific composition and chemical-physical characteristics, such as the high content of non-protein nitrogen compounds, high water activity, and neutral pH, fish are considered among the most perishable foods, as previously reported [112]. Furthermore, fish products are often still preserved with traditional methods such as refrigeration, freezing, drying, fermentation, salting, and canning, which are not always suitable to guarantee optimal conservation and prevent deterioration [113].

Refrigeration, which is one of the most widely adopted preservation methods worldwide, allows for inhibiting many pathogenic microorganisms, which are unable to grow at low temperatures. However, this technology, used alone, fails to inhibit psychrotrophic microorganisms, including pathogenic species and SSOs [72]. In addition, during refrigerated storage, several unwanted metabolites, such as trimethylamine, ammonia, biogenic amines, etc., can be formed through microbial activities [114]. For these reasons, refrigeration, used to extend the shelf life of fish products, is often used in combination with packaging, which makes it more effective [115]. However, since packaging is generally made of plastic, this combined technique poses significant environmental issues that will be addressed in the next section.

For the reasons briefly discussed here, every year, many tons of fish are lost or wasted along the entire supply chain, from capture to subsequent stages of preparation, storage, distribution, retail, and domestic consumption [62]. This waste not only leads to significant economic losses but also has a negative impact on food safety and the environment.

The scarcity of time for cooking, the desire to maximize leisure time, and social and lifestyle changes are the major factors motivating consumers to change their food consumption habits globally, with an increasing preference for convenience foods [116]. Convenience foods include “ready to eat”, “ready to heat”, and “ready to cook” foods [117]. Consumers perceive convenience foods to save time and energy, reduce stress, and minimize the effort of cleaning and food preparation. In fact, besides convenience, reduced cooking skills, physical and mental strain in preparing meals, cleaning, and waste management also play a significant role in driving consumer preference for convenience foods [116].

Although convenience foods help reduce household waste, which is particularly high for fish, they also present challenges, especially regarding preservation methods. The fishery industry must not only meet consumer demands for convenience and quick preparation but also ensure food safety, the absence of chemical additives, extended shelf life, and high sensory and nutritional quality [72]. One possible strategy to meet these sometimes conflicting consumer demands is eco-friendly packaging.

## 6. Packaging

Packaging plays a crucial role in protecting food products from physical damage and environmental factors, preserving their quality and safety, and extending shelf life to minimize food losses and waste. Beyond its primary protective function, packaging facilitates transportation and distribution, provides essential information on product composition, nutritional properties, and production methods, and includes guidelines for storage and preparation, all while serving as a marketing tool [118]. The global packaged food market was valued at approximately USD 1.9 trillion in 2020 and is projected to reach USD 3.4 trillion by 2030 [119]. Conventional, petroleum-based polymers such as polypropylene, polyester, and polyvinyl alcohol have dominated the packaging industry due to their favorable mechanical and barrier properties. However, their non-biodegradable nature has led to significant environmental concerns, as these materials persist in ecosystems and landfills, contributing to pollution and posing risks to human health [120,121]. As a response, the food industry has increasingly shifted away from direct waste disposal in favor of reuse and recycling strategies. Landfilling organic waste, including packaging waste, can result in methane emissions up to 2000% higher than other disposal methods, significantly exacerbating global warming [122]. In this context, sustainable packaging solutions—often referred to as green packaging, eco-friendly packaging, or sustainable packaging—are gaining attention [123,124]. The primary objective of sustainable packaging is to incorporate materials that minimize waste generation, both during production and after disposal, without compromising the protective function of the packaging [125]. In response to increasing consumer demand for fresh, minimally processed foods with preserved nutritional and sensory qualities, recent research has focused on the development of edible coatings and films as innovative packaging alternatives [126]. In particular, the application of biodegradable and edible packaging solutions in the seafood industry, including for *Dicentrarchus labrax* and *Sparus aurata*, represents a promising strategy to enhance product shelf life while reducing environmental impact, aligning with circular economy principles in Mediterranean aquaculture.

### 6.1. Active and Intelligent Packaging

Active packaging monitors the quality of the food product or its environment to quantify shelf life [127]. Referring to the European legislation regarding active packaging, specifically the 107/89 EC directive on food additives [128], active packaging could “modify the composition or organoleptic properties of the food only if the modifications comply with the community provisions applicable to foods”. Among the types of active packaging, we find releasing packaging and absorbent packaging. In the first case, the polymers that make up the edible coating are made up of various functional and active compounds which are, during the storage period, gradually released into the food, thus generating an antagonistic action against its deterioration, while with absorbent packaging there is an interaction with the food, where the coating absorbs the compounds of the product without releasing its active ingredients and components into the latter. With both types of coating, the nutritional and organoleptic quality of the packaged product is improved [129]. Moisture absorbers, carbon dioxide emitters, antioxidants, and antimicrobial release and containment systems are used to produce active packaging. The addition of antimicrobial additives in food packaging promotes a reduction in food spoilage due to microbial contamination [130]. For the active packaging of fish products, systems are adopted that absorb oxygen, emit CO₂, use humidity regulators, and release antimicrobials and antioxidants.

Reale et al. [72] demonstrated in their study how the use of chitosan can be useful for the preservation of sea bass, as its antimicrobial action is exploited, safeguarding the sensorial and organoleptic characteristics of the food product. Mainly, the antimicrobial action has been demonstrated against *Pseudomonas* spp. and *Brochothrix thermosphacta*, also demonstrating that thanks to the structural characteristics of chitosan, it can be used as a constituent of active edible films. Fu et al. [131] showed how the use of gallic acid-modified agarose in the preparation of active packaging for seafood products should not exceed a concentration of 2%, as it does not allow significant improvement in the microbiological characteristics of the seafood product but still positively affects preservation. Exceeding the concentration of 2% causes an increase in the viscosity of the modifying agarose gel, disfavouring the formation of the coating. Gallic acid has remarkable antioxidant and antimicrobial properties, as demonstrated in several studies [80,132]. The use of these substances with antimicrobial activity has been demonstrated by countless studies conducted by Maleki and Mohsenzadeh [133], who used a film with carboxymethyl cellulose and titanium dioxide nanoparticles with fennel essential oil for fish samples. Eshaghi et al. [134] demonstrated that films containing titanium dioxide nanoparticles in combination with dill essential oil can inhibit the growth of microorganisms in fish products, prolonging their shelf life. In fact, they observed that in fish preserved with this technique, on the twelfth day of storage, the microbial load of psychrotrophic bacteria was approximately 7 log CFU/g lower compared to the control sample.

Intelligent packaging is defined as “intelligent” because it implies a switching function that varies based on the change that occurs due to external or internal stimuli. This kind of packaging can provide information regarding the quality of food throughout the food chain [135]. Unlike active packaging, smart packaging communicates any changes directly to the consumer via a device inside it [136]. Intelligent packaging is a casing capable of controlling the conditions and quality of products during storage, transport, and home preservation using sensors and indicators. It is able to detect and record changes in the food product and/or its environment, thus providing warnings on problems that may occur during the storage or transport phase. There are different types of time-temperature indicators, gas detectors, and freshness indicators [137]. They are defined by Regulation (EC) no. 1935/2004 [138] as “materials and objects that control the conditions of the packaged food product or its environment”. Intelligent packaging can inform the consumer and operators on the state of conservation and quality of the food [139]. Innovations in the field of intelligent packaging mainly concern the use of natural biodegradable polymers obtained from proteins, polysaccharides, and lipids, but also from biomass or other animal, plant, or possibly microbial sources [140].

The indicators used in the packaging of fish products can be useful for food sector operators to understand whether the fish is fresh or not [141]. The food industry has adopted, with the use of intelligent packaging, rapid systems for detecting microbial degradation products in packaged fish [142]. An example is the non-invasive colorimetric system adopted by Pacquit et al. [143], thus allowing real-time monitoring of deterioration in cod samples. The preparation made it possible to insert the sensors (pH-sensitive dyes such as bromocresol green) inside the polymeric matrix, which, when the optimal storage conditions changed, presented a color visible to the naked eye from yellow to blue. Tavassoli et al. [144] produced intelligent packaging using anthocyanins obtained from red poppies. The latter allowed us to verify any deterioration of the fish by changing the color of the packaging from yellow to grey after approximately 72 h. They also showed a good inhibitory capacity against *E. coli* and *S. aureus*, but further studies must be carried out to allow their industrial use. Chung et al. [145] have developed a new intelligent packaging containing a chip that uses intelligent sensors to quantify the freshness of fresh fish. These sensors specifically quantify the quantity of H2S or NH3 produced in the fish during the storage period. They demonstrated that if the packaged fish is stored at room temperature, the concentrations of the two gases cause a sudden decay of the fish with a change in odor and color compared to if it is stored at refrigeration temperatures.

### 6.2. Edible Packaging

A suitable strategy for ensuring food protection and safety is the use of edible packaging, classified as primary packaging, composed of comestible components, such as polysaccharides, proteins, and lipids. Polysaccharides and proteins, which are hydrocolloids, have interesting mechanical and structural properties, while lipids are used to reduce water transmission thanks to their hydrophobicity. Among the polysaccharides, cellulose, starch, gums, and chitosan are the most used, while casein, zein, gluten, and gelatine are some of the common proteins used to produce edible packaging. Lipids instead are represented by different waxes, triglycerides, fatty acids, and so on. These ingredients can be used individually or together to enhance the positive properties of each component [146]. The use of edible packaging has been promoted as a safe, inexpensive, easy, and sustainable way to extend the shelf life of different types of foods while reducing packaging waste [147]. Such technology extends the shelf life of foods by protecting them from a variety of physical and biological attacks, including microbial contamination [148], and its use can also improve product quality, giving nutritional benefits [149,150]. The food industry, with the term edible packaging, refers to both edible coatings and edible films. The former is a thin layer of edible material directly applied in a liquid form on the surface of the food. At the same time, the film is stand-alone, preformed wrapping material applied as a food package [151]. One of the advantages of edible coatings and films is that among the “basic/main” ingredients represented by the biopolymers, the formulations can be enriched with functional components, including antimicrobials or antioxidants [152], which can be obtained from different sources.

A substance is defined as “functional” by the EFSA [153], which, after an in-depth study, includes it in the appropriate list. There are various bioactive substances naturally present in foods, such as vitamins, probiotics, and prebiotics, with positive actions at the gastrointestinal level [154,155]; or polyphenols, flavonoids, and carotenoids can help in degenerative health conditions such as carcinomas, Parkinson’s, cardiovascular problems and many others [156,157]. Equally interesting is the use of by-products derived from food processing because they still have interesting compounds that can be used to replace synthetic food additives [14,158].

The European Directive 2008/98/EC [159], article 5 defines by-product “a substance or object deriving from a production process whose primary purpose is not the production of that article may not be considered waste within the meaning of Article 3, point 1” (waste: any substance or object which the holder discards or intends or is obliged to discard). Therefore, given the high quantities of waste obtained from the food sector, it is necessary to think about reusing the by-products obtained to obtain novel foods or to preserve products already on the market.

For example, grape waste, peel, and seeds from tomato, soybean, and walnut husks are rich in polyphenols, which play a fundamental role in some food preparations. In the plant environment, polyphenols counteract the action of pathogenic microorganisms and prevent the formation of free radicals, thanks to their further ability to oxidize [160]. Moreover, antioxidants are described in Annex I of Reg. 1333/2008/EC [161], as substances capable of prolonging the shelf-life of food, protecting it from phenomena of color variation deterioration caused by oxidation. For this reason, the use of polyphenolic compounds could be useful in extending the shelf-life of the food product, as well as the use of antimicrobial substances. The antimicrobial mechanism of plant substances against pathogens can be attributed to different pathways of action, such as rupture of the cell wall, alteration of the permeability, and fluidity of the cell membrane with consequent leakage of the cellular contents [162]. Inhibition of pathogen metabolism through the tricarboxylic acid (TCA) cycle, blockade of adenosine triphosphate (ATP) synthesis, and production of reactive oxygen species (ROS) can affect pathogen survival [163,164].

Based on the above definitions, numerous food by-products have been successfully utilized to extract functional, antimicrobial, and antioxidant compounds for incorporation into edible packaging (Table 1). Notably, several of these by-products have been effectively applied to enhance the packaging and shelf life of *Dicentrarchus labrax* (sea bass) and *Sparus aurata* (sea bream), demonstrating their potential to preserve quality, extending freshness, and reducing microbial contamination in Mediterranean aquaculture products.

An example of natural compounds that exhibit the mentioned properties are essential oils obtained from various plant parts (leaves, flowers, and peels). The use of essential oils in both Europe and the US is legislatively regulated by several regulations, and the legal aspect of their use in foods is divided into two main categories: the first concerns the use of essential oils as a food ingredient or nutrient, while the second pertains to their incorporation into food packaging materials [161]. The food industry must adhere to the legislation of the country of production, which may vary by region. Of particular importance is the recognition of some essential oils as food additives, such as rosemary extract, as outlined in Regulation (EU) no. 1130/2011 of the European Commission [172]. When essential oils are used in food packaging, it is necessary to comply with Regulation (EC) 1935/2004 [138], which governs materials intended to come into contact with food, as well as Regulation (EC) 2023/2006 [173], which sets guidelines for good manufacturing practices. The Food and Drug Administration (FDA) in the USA has identified compounds such as linalool, eugenol, vanillin, and limonene as approved flavoring agents for food applications [174]. Research in this area has demonstrated that edible coatings and films made from the aforementioned biopolymers, incorporated with various bioactive agents (often derived from natural sources or food by-products), exhibit effective and beneficial functional properties. These coatings help reduce moisture loss, slow respiration processes, and preserve the integrity of the food, ultimately extending the shelf life of products like *Dicentrarchus labrax* (sea bass) and *Sparus aurata* (sea bream). The use of essential oil-based coatings for these fish species has proven effective in maintaining quality and physical attributes, reducing microbial contamination, and prolonging their freshness.

## 7. Use of Citrus By-Products for Safety and Shelf Life of Fish and Fish Products

Citrus is one of the world’s most important fruit crops, with global production estimated by FAO at 158,490,986 million tons in 2020, of which about 30 million tons will be for juice production [175]. From the products obtained from citrus processing (juices, pastes, jams, etc.), high amounts of by-products are obtained mainly for livestock feed but also to produce bioethanol and substances with high antimicrobial activity. The annual value of by-products and waste worldwide is around 15 million tons [176]. The by-products of citrus fruits following juice production are the peel consisting of albedo and flavedo, pulp, and seeds [177]. The reuse of citrus by-products allows the extraction of essential oils from solid waste, while liquid waste can be used to produce enzymes. All this reduces the environmental impact and favors the economic and socio-economic aspects, making it sustainable [178]. The albedo, which represents the internal layer of the peel, is made up of pectin natural hydrocolloids with a gelling, stabilizing, and emulsifying action. Furthermore, it has a number of essential oils that varies based on the type of extraction and variety of the plant [179], as well as various compounds such as soluble sugars, amino acids, fiber, organic acids, lipids, and vitamins [180]. Albedo, to date, has been studied and proposed as a source of fiber and for its emulsifying capabilities as a replacement for animal fats in fresh meat and fermented meat also using well selected microorganisms [181,182]. Essential oils consist of D-limonene, α-terpinolene, α-pinene, citronellol, β-citronellol, and other compounds [183]. As mentioned previously, soluble pectins are the most present component within the albedo, but subsequently, we also find soluble sugars such as glucose, fructose, sucrose, cellulose, and hemicellulose in different quantities [184,185].

**Figure 7:**
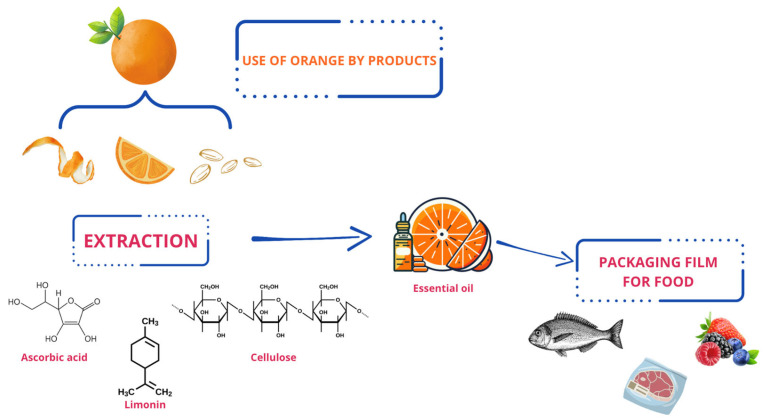
Extraction of compounds from orange by-products and use in food packaging. Use of Citrus Peel and Essential Oils to Produce Edible Coating for Fish Preservation.

Essential oil obtained from citrus peel has well-known antimicrobial action [186] and has a high concentration of monoterpene hydrocarbons, of which limonene is the most present component (Figure 7) [187]. Finally, flavonoids such as hesperidin can be obtained from the peel, which is heavily used in the production of dietary supplements as it has several beneficial actions for the body, such as antioxidant, antiallergic, antimicrobial, and antitumor action [188]. As a result of obtaining the various by-products treated above, there are additional processing residues that can be exploited in processes for energy production, thus furthering the concept of circular economy [189].

Essential oils obtained from vegetable by-products, particularly citrus peel waste, are increasingly explored as food additives due to their well-documented antimicrobial and antioxidant properties [190]. The chemical composition of citrus peel essential oils is highly complex, and while their antimicrobial mechanisms are not yet fully elucidated, their activity is believed to be largely attributed to their hydrophobic nature. The hydrophobic compounds in essential oils interact with the lipid components of microbial cell membranes, leading to increased permeability, subsequent leakage of intracellular material, and eventual cell lysis [191,192]. In seafood preservation, essential oils can be applied directly to fish products or incorporated into micro- and nano-emulsions. However, when applied in high concentrations directly onto fish, they may negatively impact sensory attributes, rendering the product unacceptable to consumers [193]. To mitigate these drawbacks, the incorporation of essential oils into micro- and nano-emulsions, particularly in the form of edible coatings, has emerged as a promising strategy. This approach enhances bioavailability and kinetic stability due to the small particle size, facilitating better dispersion and controlled release of bioactive compounds [194]. Several studies have demonstrated that essential oil-based edible coatings improve the sensory and storage characteristics of fish fillets [165,195]. Among citrus-derived essential oils, those extracted from orange peel exhibit significant antimicrobial activity against foodborne pathogens such as *Listeria monocytogenes*, *Salmonella* spp., *Staphylococcus aureus*, *Escherichia coli*, and *Bacillus cereus* [196]. This antimicrobial potential is largely attributed to the presence of flavonoids and a high concentration of volatile compounds, particularly monoterpene hydrocarbons. The effectiveness of edible coatings containing essential oils depends on various factors, including the concentration of the essential oil, which influences the thickness of the coating. This, in turn, affects both the mechanical properties of the polymer matrix and the antimicrobial efficacy of the coating [197,198]. Moreover, the addition of essential oils reduces the hydrophilicity of the edible coating, as demonstrated by Agdar GhareAghaji et al. [199], who observed a decrease in moisture absorption and water permeability within the film. Several studies have reported the effectiveness of essential oil-based coatings in extending the shelf life of fish species. For instance, a chitosan-based edible coating enriched with lemon extract extended the storage period of rainbow trout (*Oncorhynchus mykiss*) fillets up to nine days [167], a finding corroborated by Sabu et al. [200] in tuna meat. Kilinç et al. [201] formulated edible coatings derived from citrus peel waste, particularly from orange and lemon, in combination with xanthan, locust bean gum, and carrageenan. When applied to rainbow trout fillets and squid rings, these coatings significantly enhanced shelf life, improved sensory attributes, and reduced microbial contamination. Similarly, Farahmandfar et al. [202] found that essential oils from *Citrus sinensis* (L. Osbeck) exhibited stronger antimicrobial activity against Gram-positive bacteria (*L. monocytogenes*, *S. aureus*) than against Gram-negative bacteria, likely due to differences in cell wall structure. Gram-positive bacteria possess a thick peptidoglycan layer, which does not act as a permeability barrier, whereas the outer membrane of Gram-negative bacteria, rich in porins, restricts the penetration of antibacterial compounds. Conversely, Yi et al. [186] demonstrated that essential oil extracted from Nanfeng mandarin exhibited broad-spectrum antimicrobial activity against both Gram-positive and Gram-negative bacteria, as well as molds. The application of essential oil-based edible coatings in the preservation of *Dicentrarchus labrax* and *Sparus aurata* fillets represents a promising strategy for extending shelf life, reducing microbial contamination, and maintaining sensory attributes. Future research should focus on optimizing formulation parameters to enhance coating adhesion, antimicrobial effectiveness, and consumer acceptability while ensuring that these biopreservation techniques align with sustainable and circular economy principles in Mediterranean aquaculture.

## 8. Conclusions

This review highlights the importance of sustainable innovations in the supply chain management of *Dicentrarchus labrax* (European sea bass) and *Sparus aurata* (gilthead sea bream), two key species in Mediterranean aquaculture. It stresses the need to integrate circular economy principles and biotechnological solutions to address challenges in production, processing, and packaging, which are in line with the 2030 Agenda for Sustainable Development. Integrated Multi-Trophic Aquaculture (IMTA) systems offer a promising strategy for enhancing sustainability and waste reduction, though challenges such as complexity and costs remain. Regulatory frameworks, including the FAO Roadmap and the European Commission’s Strategic Guidelines for 2020–2030, supported by the European Maritime, Fisheries, and Aquaculture Fund (EUR 6.1 billion), aim to promote sustainable aquaculture practices. Advances in biopreservation, such as antimicrobial films from plant-derived antimicrobials and citrus by-products, show potential for reducing food loss and packaging’s environmental impact. These innovations support SDGs related to food security, poverty reduction, human health, and environmental conservation. To realize the potential of Mediterranean aquaculture, addressing perishability, food loss, and the environmental impact of conventional methods is crucial. Transitioning to a circular supply chain requires integrated policies, investments, and stricter regulations, along with strengthening ecolabeling frameworks, promoting consumer awareness, and fostering collaborations across sectors. Economic incentives can further support responsible aquaculture systems. In conclusion, a holistic approach is essential for achieving sustainability in Mediterranean aquaculture and can serve as a model for other regions. Future research should focus on overcoming barriers and increasing consumer acceptance of sustainable seafood.

## Figures and Tables

**Table 1:** Composition of edible films used for fish products and results of their application.

Film Composition	Sample	Storage (°C)	Results	References
Zein, orange peel extract	Fish fillets	4	Inhibition of the microbial population and lipid oxidation, resulting in shelf-life extension.	[165]
Pectin	Sea bream fillets	4	Inhibition of the microbial population	[166]
Chitosan and lemon extract	Rainbow trout fillets	4	Increase of the storage period	[167]
Pectin, gelatin, and HPMC	Chilled gilthead sea bream (*Sparus aurata*) fillets	2	Increase of the storage period	[168]
Chitosan and garlic essential oil	Shrimp meat	4	Antimicrobial activity	[169]
Chitosan	Smoked sea bass (*Dicentrarchus labrax*)	4	Increase of the storage period	[170]
*Satureja thymbra* essential oil	Sea bream fillets	0	Antimicrobial and antioxidant effects. Increase of the storage period	[171]
